# Rho-associated kinase signalling and the cancer microenvironment: novel biological implications and therapeutic opportunities

**DOI:** 10.1017/erm.2015.17

**Published:** 2015-10-28

**Authors:** Venessa T. Chin, Adnan M. Nagrial, Angela Chou, Andrew V. Biankin, Anthony J. Gill, Paul Timpson, Marina Pajic

**Affiliations:** 1 The Kinghorn Cancer Centre, Cancer Division, Garvan Institute of Medical Research, 384 Victoria St, Darlinghurst, Sydney, NSW 2010, Australia; 2 The Department of Medical Oncology, Crown Princess Mary Cancer Centre, Westmead Hospital, NSW, Australia; 3 Anatomical Pathology, Sydpath, St Vincent's Hospital, Sydney, Australia; 4 Department of Surgery, Bankstown Hospital, Eldridge Road, Bankstown, Sydney, NSW 2200, Australia; 5 Wolfson Wohl Cancer Research Centre, Institute of Cancer Sciences, University of Glasgow, Garscube Estate, Switchback Road, Bearsden, Glasgow, Scotland G61 1BD, UK; 6 Department of Anatomical Pathology, Royal North Shore Hospital, St Leonards, Sydney, NSW 2065, Australia; 7 University of Sydney, Sydney, NSW 2006, Australia; 8 Faculty of Medicine, St Vincent's Clinical School, University of NSW, Australia

## Abstract

The Rho/ROCK pathway is involved in numerous pivotal cellular processes that have made it an area of intense study in cancer medicine, however, Rho-associated coiled-coil containing protein kinase (ROCK) inhibitors are yet to make an appearance in the clinical cancer setting. Their performance as an anti-cancer therapy has been varied in pre-clinical studies, however, they have been shown to be effective vasodilators in the treatment of hypertension and post-ischaemic stroke vasospasm. This review addresses the various roles the Rho/ROCK pathway plays in angiogenesis, tumour vascular tone and reciprocal feedback from the tumour microenvironment and explores the potential utility of ROCK inhibitors as effective vascular normalising agents. ROCK inhibitors may potentially enhance the delivery and efficacy of chemotherapy agents and improve the effectiveness of radiotherapy. As such, repurposing of these agents as adjuncts to standard treatments may significantly improve outcomes for patients with cancer. A deeper understanding of the controlled and dynamic regulation of the key components of the Rho pathway may lead to effective use of the Rho/ROCK inhibitors in the clinical management of cancer.

Cancer is one of the leading causes of death worldwide, accounting for 8.2 million deaths in 2012 (Ref. 1). Although therapies for advanced stage malignancy are improving, the therapeutic options for patients are limited and often inadequate. In general, efficacy of chemotherapeutic agents is limited by adverse effects caused by their activity on normal tissues. Therefore, adjunctive treatments which specifically improve the delivery of cytotoxic therapies to the tumour may be of high value. Further, the efficacy of adjunctive therapies needs to be examined with regard to the effects on both tumour cells and the surrounding microenvironment.

The Rho/Rho-associated coiled-coil containing protein kinase (ROCK) signalling pathway plays a critical role in a range of diseases including those of the central nervous system and the cardiovascular system (e.g. spinal cord injury, vasospasm, hypertension, atherosclerosis and myocardial hypertrophy) (Refs 2, 3, 4). In cancer, over-expression of ROCK induces migration and invasion *in vitro* and *in vivo* (Refs 5, 6). Its involvement in cellular proliferation, cell shape and motility, tumour progression and metastasis (Ref. 7) make it an attractive target in cancer medicine. However, the full potential of ROCK inhibitors as anti-cancer therapies may not have been fully examined. The effects of the Rho/ROCK pathway on the vascular system have been extensively studied in the treatment of vascular disorders. Inhibition of Rho signalling within the hypoxic and abnormal tumour vasculature may lead to an improved anti-tumour efficacy of cytotoxic agents through the normalisation of the vascular supply to tumours (Ref. 8). Moreover, the effects of ROCK inhibition on other key components of the tumour microenvironment, including activated (myo)fibroblasts, immune cells and extracellular matrix (ECM), may have an additional therapeutic value (Refs 9, 10, 11). This review summarises our current understanding of the diverse and complex roles of aberrant Rho/ROCK signalling in tumour development and progression, highlighting new avenues for the utilisation of ROCK inhibitors as anti-cancer therapy, increasingly in the context of modulating the tumour microenvironment.

## Key components of the Rho/ROCK pathway

**Figure 1.**
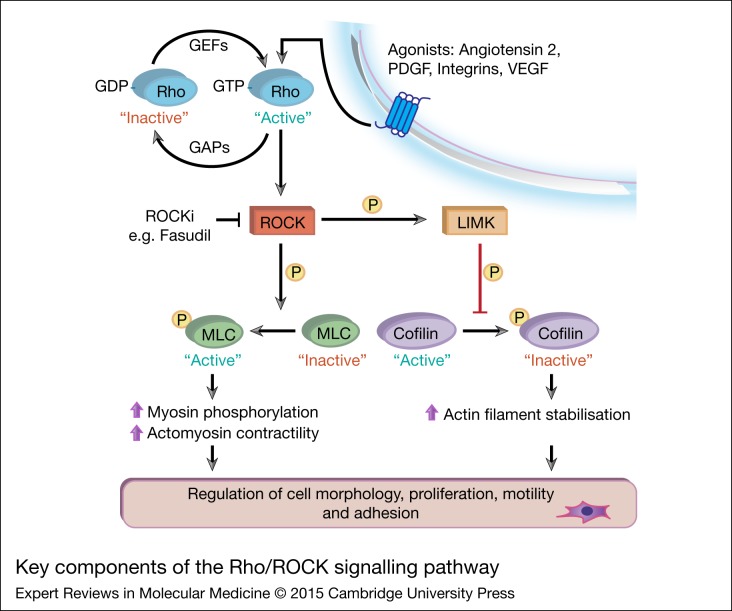
Key components of the Rho/ Rho-associated coiled-coil containing protein kinase (ROCK) signalling pathway. Various extracellular stimuli (growth factors and hormones) bind to cell membrane receptors, which subsequently act upon guanine-nucleotide-exchange factors (GEFs) and GTPase-activating proteins (GAPs) to regulate activation of Rho GTPase proteins. Once in its GTP-bound ‘active’ state, Rho GTPase binds to ROCK (ROCK1/2) to stimulate key downstream effectors (Refs 7, 12, 21). ROCK-mediated phosphorylation of myosin light-chain (MLC) promotes phosphorylation of myosin and increased actomyosin contraction. Activation of LIMK by ROCK leads to phosphorylation and inactivation of the actin-depolymerising protein cofilin, altering actin filament organisation. Collectively, activation of key downstream effectors of Rho causes changes in motility, proliferation and other essential cellular processes.

The Rho family of small GTPases regulate a diverse array of cellular processes, including cytoskeletal dynamics, cell polarity, membrane transport and gene expression, which are integral for the growth and metastatic potential of cancer cells (Ref. 7). The three best characterised members of this family are Rho (A, B and C), Rac (1, 2 and 3) and Cdc42 (Ref. 7). They cycle between a GTP-bound active state and GDP-bound inactive state which is mediated by guanine nucleotide exchange factors (GEFs) and GTPase-activating proteins (GAPs), as illustrated in Figure 1 (Refs 12, 13). In their active state, they act on one of over 60 downstream targets which include Rho-associated coiled-coil containing protein kinase (ROCK), mDia (Ref. 14), serine/threonine p21-activating kinases 4-6 (Ref. 15), Par6 (Ref. 16) and Wiskott-Aldrich Syndrome Protein (Ref. 17). In addition, through interaction with various well characterised pathways, including the phosphoinositide 3-kinase, focal adhesion kinase, Src, LIM domain kinase (LIMK) and mitogen-activated protein kinase/Erk protein networks, Rho GTPase activation ultimately leads to actin cytoskeleton remodelling, increased cell motility, changes in proliferation and cell survival (Refs 10, 18, 19, 20). ROCK, a downstream effector of Rho, phosphorylates MYPT1, the targeting subunit of myosin phosphatase, resulting in decreased myosin phosphatase activity and thereby increased phosphorylation of the regulatory myosin light-chain 2 (MLC2) protein (Ref. 21). Both ROCK/MYPT1/MLC2 and ROCK/LIMK/cofilin signalling axes are heavily involved in stress fibre assembly, cell adhesion and motility (Fig. 1). Further, the ROCK family contains two members, ROCK1 and ROCK2, which share 65% overall identity and 92% identity in the kinase domain (Ref. 22) and are thus believed to also share more than 30 immediate downstream substrates, including MYPT1, MLC, and LIMK (Ref. 7). Some differences in the activation of specific isoforms of ROCK have also been reported. For example, induction of pressure overload cardiac hypertrophy in mice leads to elevated ROCK1, but not ROCK2, expression (Ref. 22) and specific activation of the Rho/ROCK1/c-Jun N-terminal kinase (JNK) signalling in hypertrophic cardiomyocytes (Ref. 23). Similarly, ROCK2 has been implicated as the relevant isoform in a mouse model of acute ischaemic stroke (Ref. 24). Finally, emerging evidence suggests potential distinct roles of ROCK1 and ROCK2 in regulating stress-induced actin cytoskeleton reorganisation and cell detachment in mouse embryonic fibroblasts (Ref. 25) and migrating neurons (Ref. 26).

Moreover, ROCK can be effectively targeted by (non-isoform) specific inhibitors including Y-27632, fasudil and new generation compounds, which prevent activation of ROCK by competing with ATP for binding to the kinase (Refs 27, 28, 29). Interestingly, fasudil has been shown to be safe for use in humans for the treatment of cerebral vasospasm with an acceptable side effect profile, making it an attractive drug for clinical study (Ref. 30).

## Exploring the effects of inhibiting Rho/ROCK in cancer: the pre-clinical evidence

Numerous studies have thus far investigated the therapeutic efficacy of Rho/ROCK inhibition in *in vitro* and *in vivo* models of cancer (Table 1, (Refs 5, 28, 29, 31, 32, 33, 34, 35, 36, 37, 38, 39, 40, 41, 42, 43, 44, 45, 46, 47, 48, 49, 50, 51, 52, 53, 54, 55, 56, 57, 58). As summarised in Table 1, blocking Rho/ROCK signalling in cancer cells can effectively reduce cellular proliferation, invasion and angiogenesis *in vitro* and reduce tumour growth and metastasis formation *in vivo*. Interestingly, the effects on proliferation are heterogeneous, with several studies reporting no effect at all (Refs 21, 28, 39, 42, 46, 49), one study demonstrating an anti-proliferative effect when fasudil was used at a supraphysiological concentration (Ref. 38) and several more recent studies suggesting marked effects on cell growth (Refs 29, 31, 43, 52, 56) that can be further enhanced when ROCK inhibition is combined with chemotherapy (Refs 35, 43). Further, when efficacy of ROCK inhibitors was examined in the context of tumour cell motility, migratory and invasive characteristics, more consistent findings were observed across a variety of cancer models examined (Refs 5, 28, 39, 42, 49). Several groups have also shown that inhibition of ROCK and its stimulated signalling might prove to be a promising strategy for restraining tumour progression *in vivo*, for example by slowing down primary tumour growth (Refs 45, 52, 55) and formation of metastases (Refs 37, 48, 49, 51, 56). The potential differences observed between the *in vitro* [two-dimensional (2D) observations] and *in vivo* findings may be partially explained by the different models examined, origin of the inhibitors used (Table 1), or the critical role RhoA plays in cellular invasion and metastasis (Ref. 59). Perhaps, this discrepancy could also be more reflective of the complex involvement Rho/ROCK has in cellular processes in cancer that cannot be accurately recapitulated in simple 2D assays (Ref. 60). A deeper understanding of Rho/ROCK signalling activation *in vivo* is necessary to fully characterise the importance of inhibiting this pathway in cancer medicine as has recently been achieved for its prototype partner Rac GTPase (Ref. 61).

**Table 1.** The therapeutic efficacy of RhO/ROCK inhibitors (ROCKi) in various models of cancer.

Species/Cancer type	Model	Inhibitor examined	Origin of inhibitor	Effect on proliferation	Effect on invasion	Effect on angiogenesis	*In vivo* findings	Additional comments	Study
Human									
Acute myeloid leukaemia	Primary leukaemia culture	Fasudil Y-27632	Selleck Chemicals	↓	–	–	↓ Tumour (leukaemia) load *in vivo*	↑ Apoptosis ↓ Leukemic progenitors	(52)
Bladder cancer	UM-UC3, 5637	Fasudil	Asahi Kasei Pharma^a^	↓	↓	–	–	↓ Migration ↑ Apoptosis	(31)
Breast cancer	MDA-MB-231	RKI-18	In-house (129)	No effect	↓	–	–	↓ Migration and anchorage independent growth	(28)
	MDA-MB-231, SUM 1315, MCF-7	Y-27632	Sigma	↓	↓	–	No effect on primary tumour weight ↓ Formation of bone metastases	↓ Migration	(37)
	MDA-MB-231	Y-27632, ROCK shRNA	Sigma	–	↓	–	No difference in tumour volume when knockdown cells were injected into mice	↓ Migration	(36)
	MDA-MB-231	RhoA/C siRNA	Eurogentech	↓	↓	↓	↓ Tumour growth and vascularisation	–	(44)
Colorectal cancer	HCT116, HT29	Y-27632	R&D Systems	–	–	–	↓ Formation of intrahepatic metastases	↓ Migration	(51)
Glioblastoma	T98G, U87MG	Fasudil	Biaffin GmbH	↓ (100 µM)	–	↓	↓ Tumour growth		(38)
	T98G, U251	Fasudil	Chasesun Pharmaceutical	↓	↓	−	↓ Tumour growth, invasion ↑ Survival	↑ Apoptosis	(32)
	LN-18	Y-27632	Calbiochem	↓	–	–	–	–	(58)
Hepatocellular carcinoma	Li-7	Y-27632	Welfide Corporation ^a^	–	–	–	↓ Formation of intrahepatic metastases	–	(48)
	Li-7, KYN-2	Dominant negative p160 ROCK mutant	In-house	–	–	–	↓ Formation of metastases (p160ROCK mutant tumours)	↓ Cell motility (p160ROCK mutant cells)	(33)
Fibrosarcoma	HT1080	Wf-536	Mitsubishi Pharma	–	↓	–	–	↓ Migration	(41)
Melanoma	NRAS-mutant SK-MEL147, BLM	GSK269962A (ROCKi) + GSK1120212 (MEKi)	Axon Medchem Selleck Chemicals	↓	–	–	↓ Tumour growth ↑ Survival with combination therapy	↑ Apoptosis and cytostasis with ROCKi + MEKi combination	(29)
Non-small cell lung cancer	A549	Fasudil	Hongri Pharmaceutical	↓	↓	–	–	–	(57)
	95D	Fasudil	Hongri Pharmaceutical	↓	↓	–	–	↓ Adhesion	(54)
	A549	Y-27632	Sigma	↓ (Y-27632 given prior to cisplatin)	–	–	–	–	(35)
Ovarian cancer	A2780, A2780CDDP (cisplatin resistant)	Fasudil, Y-27632	Sigma	↓	–	–	–	↑ Cisplatin-induced apoptosis and growth inhibition	(43)
	Caov-3, SKOV3ip1	Fasudil	Asahi-Kasei Corporation	No effect	↓	–	↓ Tumour growth ↓ Formation of ascites (SKOV3ip1)	–	(42)
	SKOV3, OVCAR3	Y-27632, Lovastatin	Calbiochem	–	↓	–	↓ Formation of metastases when treated with Lovastatin	–	(34)
Prostate cancer	PC3	Y-27632	Sigma	↓	–	–	↓ Tumour growth ↓ Formation of lung metastases	↓ Cell motility and migration	(56)
	PC3, LNCaP	Y-27632	Yoshitomi Pharmaceutical^a^	No effect	–	↓	↓ Tumour growth ↑ Survival	↓ Migration	(46)
	PC3	Wf-536	Welfide Corporation^a^	–	–	↓	↓ Tumour growth in combination with Marimastat and/or Paclitaxel	↓ Migration	(47)
Kidney carcinoma	A-498, 769-P	ROCK1 siRNA	Invitrogen	–	↓	–	–	↓ Cell motility	(50)
Mouse									
HCC	CB0140C12	Y-27632	Welfide Corporation ^a^	–	↓	–	↓ Tumour growth ↓ Formation of metastases	↑ Apoptosis ↓ MMP-9 expression	(73)
Lung carcinoma	Lewis Lung Cancer	Wf-536	Mitsubishi Pharma	No effect	↓	↓	↓ Formation of metastases	↓ Migration	(40)
Melanoma	B16F10	H1152 Fasudil	Calbiochem Selleck Chemicals	No effect	↓	–	↓ Tumour growth ↑ Survival (both ROCKi) ↓ Pulmonary metastases (H1152)	↓ Migration ↑ Intratumoural leukocyte infiltration	(49)
	B16	Fasudil	Hongri Pharmaceutical	–	–	↓	↓ Tumour growth	↓ Migration Disrupted actin stress fibres	(53)
	B16F1	Y-27632	Sigma	↓	↓	–	↓ Tumour growth	–	(45)
	B16BL6, B16F10	Wf-536	Mitsubishi Pharma	No effect	↓	–	↓ Formation of metastases ↑ Survival when combined with Paclitaxel	–	(39)
Rat									
Hepatoma	MM1	Y-27632	Yoshitomi Pharmaceutical^a^	No effect	↓	–	↓ Formation of metastases, ascites ↓ Incidence of tumour dissemination	–	(5)
Other (Mixed)									
	MDA-MB-231 HT1080 MM1	Fasudil	Asahi-Kasei Corporation	↓	–	–	↓ Tumour formation (MDA-MB-231) ↓ Formation of lung metastases (HT1080) ↓ Peritoneal dissemination (MM1)	↓ Migration	(55)

a Indicates pharmaceutical collaboration.

## The Rho/ROCK pathway is critical in angiogenesis

Sustained angiogenesis is one of the key hallmarks of tumour progression (Ref. 62) that incorporates abnormal signalling cues from key cell types within the complex tumour microenvironment (Ref. 63). It is well documented that in response to tissue hypoxia, angiogenesis is constantly stimulated resulting in a highly abnormal vasculature (Ref. 64). These vessels are immature, tortuous, have increased permeability and lead to intratumoural hypoxia, which can mediate resistance to anti-cancer therapies (Ref. 65). Moreover, the tumour-associated angiogenic vasculature, growth-promoting trophic factors that are expressed and secreted by the endothelial cells and prolonged hypoxia can collectively drive hyper-proliferation and development of a more aggressive tumour phenotype with increased propensity to metastasise (Ref. 66). Angiogenesis is a complex process, which is largely controlled by Vascular endothelial growth factor (VEGF) and its membrane receptors. To initiate the angiogenic process, endothelial cells (ECs) lose junctional integrity and increase permeability (Ref. 67). Subsequent degradation of the basement membrane and remodelling of the ECM enables ECs to migrate, proliferate and ultimately undergo morphogenesis in order for new vessels to develop (Ref. 68).

**Figure 2.**
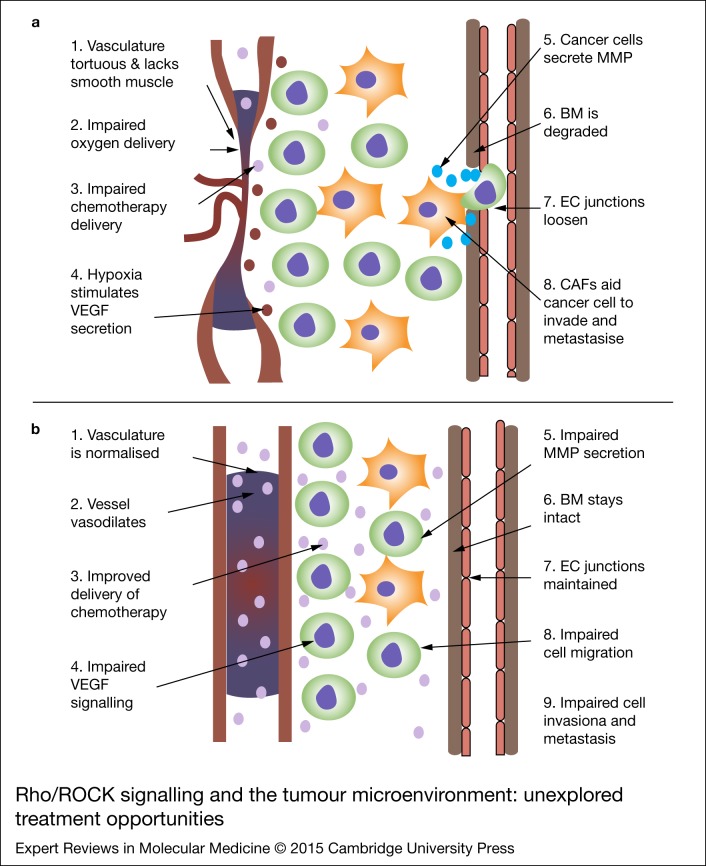
Rho/ Rho-associated coiled-coil containing protein kinase (ROCK) signalling and the tumour microenvironment: unexplored treatment opportunities. (a) Schematic illustrating key events that lead to tumour progression and metastasis. (b) In the presence of ROCK inhibitors, invasion and metastasis are impaired: the Rho/ROCK pathway as a mediator and therapeutic target of cancer metastasis. Within cancer cells, ROCK inhibitors prevent the phosphorylation of LIMK and p- myosin light-chain (MLC) which results in impaired actin-myosin filament bundling. This in turn affects cellular proliferation, morphology, adhesion, motility and gene transcription. ROCK is essential in cancer-associated fibroblasts (CAF) associated invasion and also in cell- extracellular matrix (ECM) signalling.

The Rho/ROCK pathway has been shown to be an integral part of VEGF-mediated angiogenesis and is not only implicated in VEGF signalling, but also in numerous processes necessary for angiogenesis to occur, including EC migration, survival and cell permeability (Ref. 69) (Fig. 2). It has been shown that adherin junctions between ECs need to be loosened in order for EC migration and proliferation to occur (Ref. 66). Rho/ROCK signals via p-MLC break down intracellular junctions and thereby increase vascular permeability (Ref. 70). In order for ECs to invade surrounding tissue and form new vessels, the basement membrane (BM) and ECM must be disrupted via matrix metalloproteinase (MMP) secretion (Ref. 71). Rho/ROCK activation has been shown to directly stimulate MMP-9 secretion (Ref. 72) and is also associated with increased MMP expression in tumours (Refs 73, 74). Once the BM and ECM are disrupted, EC migration and tube formation can occur. van Nieuw Amerongen et al. (Ref. 75) used human umbilical vein endothelial cells (HUVECs) to show that not only do VEGF-induced changes in the EC cytoskeleton depend on RhoA, but also that growth of human microvascular endothelial cells (hMVECs) into a fibrin matrix in response to VEGF is inhibited by Y-27632, suggesting that the Rho/ROCK pathway is necessary for ingrowth of ECs. Bryan et al. (Ref. 76) showed that disruption of the Rho/ROCK pathway inhibits VEGF-mediated changes to the cytoskeleton in ECs and also that ECs treated with Y-27632 failed to assemble into recognisable vessel structures, highlighting the importance of the Rho/ROCK pathway in vasculogenesis. Hoang and Uchida (Refs 77, 78) both demonstrated that inhibiting Rho/ROCK prevented ECs from forming organised vascular structures by suppressing cellular motility. As the Rho/ROCK pathway has been established as being critical to multiple steps in angiogenesis, many studies have attempted to elucidate the importance of its involvement in the cancer setting. Croft et al. (Ref. 79) used a conditionally active form of ROCK2 in colon carcinoma cells to show that increased ROCK signalling promoted tumour angiogenesis and tumour cell invasion *in vivo*. Using HUVEC and glioma cell co-culture techniques, Nakabayashi et al. (Ref. 38) further showed that the ROCK inhibitor fasudil suppressed tumour-induced angiogenesis and the migration of HUVEC cells through transwell plates. Moreover, the same group showed that the growth of T98G glioma xenografts was significantly inhibited when tumour-bearing mice were treated daily with fasudil (Ref. 38). ROCK inhibitors also showed significant promise as anti-angiogenic agents in additional *in vivo* models, for example Nakajima et al. (Ref. 40) showed that administration of the ROCK inhibitor Wf-536 reduced the number of spontaneous metastases and impaired angiogenesis in a Lewis lung carcinoma model. Further, Somlyo et al. (Ref. 47) showed that mice bearing xenotransplants of PC3 cells had a reduction in tumour volume and increased survival when treated with a combination of Wf-536 and Marimastat (an MMP inhibitor). ROCK inhibitors have not been evaluated in human trials to date. However, considerable clinical data exists regarding the effects of VEGF inhibitors on various cancer subtypes. Although anti-angiogenic therapies have shown variable efficacy in cancer treatment, a deeper understanding of the mechanisms of action has highlighted the potential importance of timing of administration on the anti-cancer effects. This hypothesis is an interesting new strategy to explore and test.

## Rho/ROCK inhibitors as vascular normalising agents

Clinical use of anti-angiogenic agents has generated disappointing results when used as monotherapy (Ref. 80), but more success has been had when these agents are combined with cytotoxic chemotherapy (Ref. 81). A potential explanation for this may include acquired resistance mechanisms because of continual VEGF inhibition (Refs 82, 83), intrinsic vascular heterogeneity within tumours (Ref. 84) and/or impaired drug delivery because of excessive reduction in tumour vasculature, which ultimately shifts the net balance towards hypoxia-driven rebound angiogenesis (Ref. 85). VEGF inhibition leads to increased tumour oxygenation when administered in a transient manner, a process called vascular normalisation (Refs 86, 87, 88) (Fig. 2). Exploiting this process to improve the efficacy of standard cytotoxic therapies is attractive and several pre-clinical and clinical studies have explored this concept thus far. Lee et al. (Ref. 89) demonstrated that blocking VEGF in glioblastoma or colon adenocarcinoma compensates for hypoxia-induced radiation resistance. The authors further showed that using an anti-VEGF antibody resulted in greater tumour growth delay when combined with radiation, than radiation alone. Blocking VEGF signalling was subsequently found to lead to pruning of immature vessels and generation of a morphologically ‘normalized’ vascular network within tumours, allowing deeper penetration of molecules, such as chemotherapeutics into the cancer (Ref. 88). Most recently, Coutelle et al. (Ref. 90) showed that dual targeting of VEGF and Angiopoietin-2 in addition to reducing tumour growth and sprouting angiogenesis significantly improved vascular normalisation parameters, including leakiness, hypoxia and perfusion as prerequisites for improved access for chemotherapy. Importantly, in the same study, the authors also showed for the first time, that the formation of vascular basement membrane sleeves that facilitate the rapid vascular regrowth associated with resistance to VEGF-targeting drugs can be eliminated by such dual targeting strategies. Falcon et al. (Ref. 91) similarly demonstrated that platelet-derived growth factor (PDGF) beta blockade in Lewis lung carcinoma tumours increased tumour vessel efficiency *in vivo*. Further, they found that the combination of imatinib with cyclophosphamide improved the delivery of cyclophosphamide to the tumour and the tumour burden was reduced *in vivo*.

The Rho/ROCK pathway has been specifically examined in this context: Ader et al. (Ref. 92) performed *in vivo* induction of dominant negative Rho (RhoBN19) to show that inhibiting Rho decreased tumour cell survival after irradiation and moreover, tumours had improved oxygenation and decreased vessel density. The critical aspect of optimal timing of administration of combinations involving anti-VEGF therapies and cytotoxic agents was further explored by Winkler et al. (Ref. 8). Treatment of glioblastoma xenografts with an anti-VEGF receptor (VEGFR) 2 monoclonal antibody resulted in a significant reduction in tumour hypoxia on day 2, with maximal reduction on day 5. By day 8, tumour hypoxia had started to increase. Further, radiation therapy produced a synergistic effect when given on days 4–6. This suggests that after VEGFR blockade, there is an initial increase in tumour oxygenation during which, the effects of radiotherapy are increased, but importantly with continual VEGFR blockade, the tumour becomes hypoxic again and the synergism with radiation is lost. Several randomised trials have shown that the addition of bevacizumab to chemotherapy and radiotherapy improves progression free survival in patients with central nervous system malignancies (Refs 93, 94) and a phase I trial specifically testing the vascular normalisation strategy has shown this holds considerable promise in patient care (Ref. 95). Here, patients with rectal cancer receiving neoadjuvant chemotherapy plus radiation were exposed to the VEGF inhibitor, bevacizumab. Interestingly, bevacizumab treatment led to normalisation of the tumour vasculature, increased tumour cell apoptosis and resulted in a complete pathological response in two patients (Ref. 95). Therefore, it would be interesting to examine whether Rho/ROCK pathway inhibitors may prove effective vascular normalising agents, increasing efficacy of cytotoxic therapies by modulating key components of the VEGF signalling pathway. However, the transient nature of vascular normalisation means that the window of opportunity for drug delivery is temporary, may be difficult to predict and therefore apply in the clinical setting. These issues are yet to be systematically examined.

## Rho/ROCK inhibitors as provascular agents

In addition to normalising the tumour vasculature, a provascular strategy may also be a promising treatment approach, where transient vasodilation by targeted therapy improves blood supply and exposure of tumour cells to circulating chemotherapeutics and/or sensitivity to radiation. As most vasodilators dilate both the tumour and systemic vasculature, there can be unpredictable effects on the tumour vasculature. If the tumour vessels are in series with the systemic circulation, systemic vasodilation can increase tumour blood flow, however if the tumour vasculature is in parallel, then systemic vasodilation will cause a reduction in tumour blood flow (vascular steal phenomenon) (Ref. 96). An ideal provascular agent would therefore, be one that preferentially targets the tumour vascular bed. A number of studies have shown some success with this strategy, suggesting the idea has merit. Gallez and Sonveaux (Refs 97, 98) both demonstrated the possibility of increasing tumour blood flow using vasodilators. Jordan and Stewart (Refs 99, 100) further showed that *in vivo* administration of nitric oxide not only increased tumour blood flow, but sensitised tumours to the effects of radiation. Given the critical interplay between tumour hypoxia and angiogenesis, modulation of tumour-oxygen sensing has also proven an effective strategy to improve blood flow to the tumour. A systematic review of clinical trials assessing the effects of improving tumour oxygenation to radiosensitise tumours, suggests there may be clinical benefit, finding a 23% improvement in locoregional control and a 13% improvement in overall survival (Ref. 101). In terms of improving the delivery of chemotherapy, studies by Masunaga et al. (Ref. 102) and Martinive et al. (Ref. 103) observed significant improvements in the uptake of selected chemotherapies when tumour-bearing mice were injected with nicotinamide or an endothelin-1 receptor antagonist, respectively. Most recently, Wong et al. (Ref. 104) demonstrated that treatment combining low-dose Cilengitide, an αvβ3/ αvβ5 integrin receptor inhibitor, with a calcium channel blocker, Verapamil, significantly improved efficacy of chemotherapeutic, gemcitabine, in *in vivo* models of lung and pancreatic cancer. In the same study, detailed analysis of pre- and post-treatment material revealed that the cyclical administration of the dual vascular modulator-chemotherapy combination led to increased tumour vascular function and intratumoural drug delivery while reducing hypoxia and desmoplasia in these models. Finally, by comparing the ability of capillary ECs isolated from normal versus tumour microvasculature to sense and respond to physical cues in their ECM, Ghosh et al. (Ref. 105) demonstrated that tumour-derived ECs exhibit different sensitivities to various mechanical cues *in vitro* and that these abnormal responses, which may be implicated in the loss of normal structure in the tumour microvasculature, are because of aberrant and increased Rho signalling.

With this in mind, exploration of the vasodilatory effects of ROCK inhibitors in cancer may be an interesting treatment approach. ROCK inhibitors reduce vasospasm via reduction in smooth muscle contraction and down-regulation of endothelial nitric oxide synthase, leading to their use in the treatment of ischaemic stroke (Ref. 30), with significant efficacy in reducing post stroke cerebral vasospasm and an acceptable side effect profile. Importantly, no statistically significant differences in the side effects reported by patients were observed when fasudil was compared with placebo. ROCK inhibitors have been shown to normalise smooth muscle contraction and suppress vascular lesion formation, making them a therapy of interest in hypertension, pulmonary hypertension, hypertensive vascular disease and ischaemic heart disease (Refs 3, 4). It is therefore plausible to hypothesise that Rho/ROCK inhibitors may act as provascular agents, improving tumour blood flow and increasing exposure of cells to chemotherapy and/or sensitising cells to the effects of radiation (Fig. 2). However, as outlined for vascular normalisation, the timing and dosing of provascular agents are likely to be critical in determining success and this concept is yet to be systematically examined.

## Rho/ROCK signalling within the complex tumour microenvironment

The dynamic and complex interplay between tumour cells, stromal cells and the ECM affect cancer initiation, progression, metastasis and also, chemoresistance (Refs 106, 107). Recent data indicate that carcinogenesis and tumour angiogenesis result not only from the interaction of cancer cells with ECs of various origin (as discussed above), but that surrounding ‘normal’ stromal and inflammatory cells also have a crucial role in directing the formation of the blood vessels that nourish a developing tumour (Ref. 108). In addition, loss of normal tissue homeostasis during tumourigenesis initiates a stromal remodelling cascade which leads to fibroblast activation (i.e. myofibroblasts/cancer-associated fibroblasts or CAFs) and production of biomechanically and biochemically altered ECM (Ref. 109). Increased deposition and modification of the ECM mediated through CAF-expressed biochemical signalling molecules, including Rho/ROCK, Caveolin-1, Syndecans and Hippo pathway members YAP/TAZ (Refs 109, 110) can then lead to activation of signal transduction pathways that promote tumour cell growth, proliferation and survival.

Rho GTPases have been shown to be implicit in a number of stromal processes that contribute to the invasiveness and metastatic potential of cancer cells (Refs 11, 59). It has been long understood that the presence of high density stroma in breast tissue confer an increased risk of developing breast cancer (Ref. 111). Women with high mammographic densities have increased proliferation of stromal or epithelial tissue on histological examination and this has been correlated with an increased risk of breast cancer (Ref. 111). It was further hypothesised that interactions between the stroma and epithelium ultimately lead to cancer formation (Ref. 111). In an effort to better understand this phenomenon, Lisanti et al. (Ref. 112) conducted genome-wide transcriptional profiling of low density (LD) breast fibroblasts, compared with high density (HD) breast fibroblasts, revealing differences in several key processes including stress response, inflammation, stemness and signal transduction. The authors postulated that the presence of HD fibroblasts could be considered a pre-cancerous phenotype and Rho GTPase activation (along with increased JNK1, inducible nitric oxide synthase, fibroblast growth factor receptor, epidermal growth factor receptor and PDGF receptor signalling) was identified as a key biological process in this setting (Ref. 112). Moreover, in an *in vitro* system of tumour explants embedded in collagen gels, activation of Rho/ROCK was shown to be essential for contractility-dependent collagen realignment, whereas inhibition of Rho/ROCK led to a substantial reduction of contact guidance tracks, an early step in the invasion process (Ref. 113). Goetz et al. (Ref. 114) further demonstrated that high levels of stromal Caveolin-1, an activator of Rho/ROCK signalling (Ref. 115), can initiate ECM re-organisation in the tumour and in the cancer-associated stroma, promoting metastatic behaviour in a Rho–ROCK-dependent manner. Conversely, in the same study, down-regulation of Caveolin-1 blocked Rho/ROCK activity, leading to altered ECM topography and reduced cell contractility (Ref. 114).

Further work in breast cancer has shown that breast cancer cells grown in a 3D floating matrix differentiate into tubular structures, however if the same matrix is attached to the dish, the cells do not differentiate, but proliferate and spread (Ref. 116). In the same study, differentiation could be disrupted by increasing the density of the matrix. Interestingly, it was also shown that tubulogenesis required contraction of the 3D matrix which was dependent on the Rho/ROCK pathway and that RhoA activity was down-regulated in differentiated cells (Ref. 116). Subsequently, p190RhoGAP-B was shown to mediate down-regulation of RhoA activity and inhibition of ductal morphogenesis. RhoA activity was reduced at cell-cell adhesions versus activity at cell-ECM adhesions (Ref. 117). These studies highlight the important role the Rho/ROCK pathway has in how cancer cells interact with their environment, and how this environment in turn, affects tumour cell behaviour.

The stromal compartment of tumours has long been thought to contribute to the aggressive phenotype of cancers, and CAFs have been found to provide tumour cells with proliferative and anti-apoptotic signals affecting angiogenesis and ECM remodelling. Specifically, Cadamuro et al. (Ref. 118) showed that PDGF-D plays a major role in CAF recruitment and activates Rho/ROCK to promote fibroblast migration. Further, increased palladin expression in CAFs is associated with increased growth and metastasis of pancreatic cancer cells by increasing their ability to remodel the ECM, thereby promoting tumour invasion (Ref. 119). Gaggioli et al. (Ref. 120) demonstrated that squamous cell carcinoma (SCC) cells required fibroblasts to invade into a 3D organotypic matrix. Moreover, they showed that inhibition of Rho/ROCK signalling specifically in the fibroblasts (not in the SCC cells) reduced invasion of the SCC cells. In addition, Scott et al. (Ref. 121) showed that LIMK signalling, downstream of ROCK, is required for path generation during cancer cell invasion by both leading tumour cells and stromal cells. These findings suggest that the presence of fibroblasts is necessary for cancer cell invasion and that the Rho/ROCK activation is critical in this context. Similarly, Sanz-Moreno et al. (Ref. 10) demonstrated a role for cytokine signalling through GP130-IL6ST/JAK1 in the regulation of ROCK-dependent actomyosin contraction, which drives matrix remodelling by CAFs and migration of melanoma cells. Interestingly, the ROCK-induced actomyosin contractility was found to further stimulate JAK1/STAT3 signalling, indicating that there is a self-reinforcing positive feedback loop (Ref. 10). Therefore, inhibition of Rho/ROCK signalling in this context may block both intrinsic and microenvironment-derived extrinsic signals that promote CAF-facilitated cancer invasion, and could potentially have a sustained effect by breaking the positive feedback loop.

Migration and invasion are important elements of the growth of the primary tumour, but also play a critical role in the development of metastasis. *In vivo*, cells must breach the endothelial barrier to metastasise (Refs 122, 123). The process of intercalation is where cancer cells first adhere to ECs, open the EC junctions, stimulate EC retraction and then insert into the endothelial monolayer. It has been shown that Cdc42 depletion impairs intercalation in PC3 cells and also that Cdc42, RAC1 and RhoA impair EC junction opening. Mice injected with Cdc42 depleted PC3 cells developed fewer metastases, highlighting the importance of the Rho GTPases in intercalation (Ref. 124). Collectively, these studies indicate a critical role for the Rho/ROCK pathway in modulating relevant cross-talk between tumour cells and their surrounding microenvironment, particularly in the context of driving cellular migration, invasion and metastasis (Fig. 2).

## Conclusions and the long road to clinical translation

The Rho/ROCK pathway has been a popular field of study for cancer researchers. However, despite ROCK inhibitors being demonstrated to be safe for human use, these agents have not yet been translated to the cancer clinic. These compounds have well documented effects on cellular proliferation, however their effects on cell invasion, tumour growth and metastasis appear to be more robust. Large scale cancer genome sequencing studies have revealed that mutations in the Rho GTPase family are rare (Refs 125, 126), where generally aberrant activation of this pathway occurs through overexpression of Rho GTPases or by changes in the levels of regulators of Rho activity, including increased activation of GEFs and inactivation or loss of GAPs or GDIs. Importantly, it should be noted that increased expression of Rho/ROCK signalling components may not necessarily correlate with an increase in total activity of these proteins, as this process is also tightly regulated through subcellular localisation of Rho and downstream effectors and by their interaction with key regulatory molecules (Refs 59, 61, 127). Thus, although this is an active area of research, there are currently no effective predictive biomarkers of treatment response to Rho/ROCK inhibition.

In addition to their effects on tumour cell proliferation and motility, ROCK inhibitors modulate angiogenesis and vascular tone and thus could potentially improve the delivery and efficacy of chemotherapy or other novel targeted agents (Refs 29, 34, 47). The Rho/ROCK pathway is also important in regulating the dynamic cross-talk between tumour cells and their microenvironment which may also be therapeutically exploited to inhibit metastasis formation. Finally, the therapeutic potential of ROCK inhibitors as an adjunct to cytotoxic chemotherapy is yet to be systematically examined.

As differences in the activation of the two ROCK isoforms have been reported in cardiovascular or CNS disorders, with ROCK1 implicated as the predominant mechanism for the hypotensive effects of pan-ROCK inhibitors, one could hypothesise that there may be isoform-specific regulation of cancer cell behaviour, interactions within the tumour microenvironment and control of carcinogenesis and metastasis. From this, targeting ROCK2 could potentially lead to less toxicity compared with pan-ROCK inhibition. Attempts to produce more specific and clinically suitable ROCK inhibitors are ongoing, with increased focus on isoform-specific targeting (Ref. 128). On the other hand, given that tumours are highly adaptive and rapidly acquire resistance when exposed to therapy, hitting multiple oncogenic signalling nodules or hallmarks of cancer with non-isoform selective ROCK inhibitors, may overall represent a more effective treatment strategy, as recently highlighted by Hanahan D (Ref. 63).

Further understanding of Rho signalling in the various tumour compartments will determine whether the inhibitors of this complex pathway may serve as effective treatments for newly diagnosed or recurrent tumours and will establish the optimum combinations with radiation, cytotoxic chemotherapy, and other targeted molecular compounds. Importantly, these agents may improve the delivery of chemotherapy to the tumour, perhaps enhancing efficacy, reducing the effective dose required or overcoming some mechanisms of chemoresistance.

## Research in progress and outstanding research questions

This review highlights a number of avenues for further research when examining the clinical utility of ROCK inhibitors to treat cancer. Some interesting areas of research include closely examining how the Rho/ROCK pathway is implicated in tumour stromal signalling, particularly in cancers where tumour stroma is highly prominent such as pancreatic cancer. Studying the stroma for potential biomarkers of tumour response may provide additional important insights rather than solely focusing research on the tumour itself. State of the art molecular imaging techniques such as Forster resonance energy transfer (FRET) imaging can provide relevant information into the dynamic and spatiotemporal regulation of cell signalling behaviour under physiological and disease conditions. Transgenic mice expressing Rho GTPase FRET biosensors will provide detailed knowledge of the normal physiological roles this pathway plays at the cellular level. In addition, crossing these mouse strains with other disease models will allow us to examine, in an intact 3D system, how this pathway is involved in cancer initiation, progression, chemotherapy responsiveness and chemoresistance mechanisms. This knowledge will allow further biomarker development, examination of the effects of ROCK inhibition in primary versus metastatic lesions and in pre-cancerous lesions.

ROCK inhibitors have yet to make an appearance in the clinical setting to treat patients with cancer. They have been shown previously to have an acceptable side effect profile when used to treat post cerebrovascular accident vasospasm, but these patients had a short, continuous infusion and were monitored in intensive care. Patients with cancer will need long term exposure and ideally, take an oral preparation. Before trials examining the anti-cancer effects of these drugs can be planned, further phase I studies need to be conducted to determine the most appropriate dosing schedule and with chronic dosing in mind. Protracted infusion with a pump, such as that used for 5-fluorouracil in the oxaliplatin, 5-fluorouracil and folinic acid (FOLFOX) chemotherapy combination for colon cancer is possible, but could potentially considerably increase the cost of the treatment as well as patient morbidity. Hypotension is the most predictable side effect for patients (Ref. 30), and it may mean that elderly patients would be less likely to tolerate this drug well, which could be an issue in the management of pancreatic cancer. In our pre-clinical trials laboratory, our early data indicate that mice are able to tolerate a daily, oral preparation of a ROCK inhibitor and this is associated with measurable anti-tumour effects. Further systematic *in vivo* studies are needed to exactly predict the optimal sequence of administration of these drugs in conjunction with chemotherapy or other targeted therapeutics.

An interesting challenge remains in determining which Rho GTPase family members are the most promising druggable targets and how significant the beneficial effects of targeting this signalling network, in combination with other targeted agents and/ or conventional chemotherapeutics, will be. Further studies are necessary to accurately ascertain the effects this pathway has in cancer and in cancer stroma and if possible, identify potential biomarker(s) of response. Refining exactly which patients are most likely to benefit and which combinations dosing schedules are most effective is the key goal for further research.
